# Temporal Trend in the Coexistence of Risk Behaviors for Noncommunicable Diseases in Brazil: 2009–2019

**DOI:** 10.5888/pcd20.220296

**Published:** 2023-04-06

**Authors:** Thaís Cristina Marquezine Caldeira, Luiza Eunice Sá da Silva, Taciana Maia de Sousa, Marcela Mello Soares, Rafael Moreira Claro

**Affiliations:** 1 Postgraduate Program in Public Health, Medical School, Federal University of Minas Gerais, Belo Horizonte, Minas Gerais, Brazil; 2 Nutrition Department, Federal University of Minas Gerais, Belo Horizonte, Minas Gerais, Brazil

## Abstract

**Introduction:**

Individuals can accumulate multiple risk factors for noncommunicable diseases, increasing the chance of adverse health outcomes. We aimed to analyze the temporal trend in the coexistence of risk behaviors for noncommunicable diseases and their association with sociodemographic characteristics among adults in Brazil from 2009 through 2019.

**Methods:**

This cross-sectional study and time-series analysis was based on data collected by the Surveillance System for Risk Factors and Protection for Chronic Diseases by Telephone Survey (Vigitel) from 2009 through 2019 (N = 567,336). We used item response theory to identify the coexistence of risk behaviors (infrequent consumption of fruits and vegetables, regular consumption of sugar-sweetened beverages, smoking, abusive alcohol consumption, insufficient leisure-time physical activity). We used Poisson regression models to assess the temporal trend in the prevalence of the coexistence of noncommunicable disease–related risk behaviors and associated sociodemographic characteristics.

**Results:**

Risk behaviors that most contributed to the occurrence of coexistence were smoking, consumption of sugar-sweetened beverages, and alcohol abuse. Coexistence was more frequent among men and was inversely associated with age and education level. During the study period, we found a significant decrease in coexistence (adjusted prevalence ratio decreased from 0.99 in 2012 to 0.94 in 2019; *P* = .001), especially before 2015 (adjusted prevalence ratio = 0.94; *P* = .001).

**Conclusion:**

We found a reduction in the frequency of the coexistence of noncommunicable disease–related risk behaviors and their association with sociodemographic characteristics. It is necessary to implement effective actions to reduce risk behaviors, especially behaviors that lead to a greater coexistence of those behaviors.

**Summary**

**What is already known on this topic?**

Some risk behaviors may contribute to the coexistence of noncommunicable disease–related risk behaviors in Brazil.

**What is added by this report?**

Risk behaviors that most contributed to the occurrence of coexisting noncommunicable disease–related risk behaviors in Brazil were smoking, consumption of sugar-sweetened beverages, and alcohol abuse. Noncommunicable disease–related risk behaviors decreased from 2009 through 2019. Coexistence of risk behaviors was more frequent among men than women and was inversely associated with age and education level.

**What are the implications for public health practice?**

It is necessary to implement effective actions that reduce noncommunicable disease–related risk behaviors, especially behaviors that increase the coexistence of risk behaviors.

## Introduction

Noncommunicable diseases (NCDs) are nontransmissible diseases often of long duration that result from a combination of genetic, physiological, environmental, and behavioral factors. Currently, NCDs cause premature death, loss of quality of life, and substantial economic effects worldwide (1). They accounted for 71% (41 million) of global deaths in 2016 (1). In Brazil, NCDs that year accounted for 74% of all deaths, with cardiovascular diseases, cancers, chronic respiratory diseases, and diabetes being the most prevalent (1). Four modifiable behavioral risk factors are especially involved in their etiology: inadequate diet, smoking, alcohol consumption, and physical inactivity. In 2017, this group of behavioral risk factors accounted for more than 20 million deaths and 36.5% of all disability-adjusted life years lost worldwide (2).

The World Health Organization recommends reducing risk behaviors for the primary prevention of NCDs (1). Monitoring the frequency of these risk behaviors is essential for the guidance and appropriate design of health strategies.

Although NCD risk behaviors are frequently studied in isolation, they can coexist in the same person, increasing the chance of development and worsening of NCDs (3,4). In addition, temporal trend analyses of isolated behaviors do not allow an understanding of the true risk for NCDs in each population, because favorable evolutions of some behaviors (5–8) often coexist with unfavorable evolutions of others (9,10). Despite the broad availability of information on NCD risk behaviors in Brazil, few studies have applied a theoretical framework to understand the complexity involved in the coexistence of these risk behaviors and their evolution over time, often relying on the absolute number of these behaviors in each person (11). Multivariate techniques allow the identification of interactions that cannot be directly observed (12), which then can lead to the formulation of effective policies.

Our study aimed to analyze the temporal trend in the coexistence of NCD-related risk behaviors and to investigate their association with sociodemographic variables among adult residents of the 26 Brazilian state capitals and the Federal District from 2009 through 2019.

## Methods

This cross-sectional study and time-series analysis was based on data collected by the Surveillance System for Risk Factors and Protection for Chronic Diseases by Telephone Survey (Vigitel) from 2009 through 2019. Vigitel is a population-based survey consisting of annual landline telephone interviews with a representative sample of adults (aged ≥18 y) in all 26 Brazilian state capitals and the Federal District to investigate risk and protective factors for NCDs (13).

Sampling for Vigitel is conducted in 2 stages. First, 10,000 landline telephone numbers are randomly selected from telephone registers provided by the main national telephone companies. These telephone numbers are reorganized into subsamples of 200 numbers to enable better management of the progress of field work each year. The second stage consists of identifying the eligible numbers and randomly selecting 1 of the adults living in the household for the interview (13). A minimum sample size of approximately 2,000 interviews per year in each city was established, allowing us to estimate the prevalence of all indicators with a maximum error of 2 percentage points and a 95% CI (13). 

The interviews conducted by Vigitel are associated with weighting factors to correct the unequal probability of the selection of households with more than 1 landline or more than 1 adult resident, as well as to match (rake) the sociodemographic distribution of the studied population with that projected for the full population of each study site in each year (based on census data and official projections) (13). Further details on the sampling data collection process are provided in the system’s annual report (13).

### Data collection and organization

This study analyzed the coexistence of 5 NCD behavioral risk factors: infrequent consumption of fruits and vegetables, regular consumption of sugar-sweetened beverages, smoking, abusive alcohol consumption, and insufficient leisure-time physical activity. Infrequent consumption of fruits and vegetables (<5 days/week) and regular consumption of sugar-sweetened beverages (≥5 days/week) were defined on the basis of questions about the frequency of consumption: “How many days a week do you usually eat [name of food group]?” The consumption of fruits and vegetables on fewer than 5 days per week was classified as infrequent consumption. The consumption of sugar-sweetened beverages on more than 5 days per week was classified as regular consumption. Smoking was identified by the affirmative answer to the question “Currently, do you smoke?” Abusive alcohol consumption (≥4 doses on a single occasion in the past 30 days for women, and ≥5 for men) was identified through an affirmative answer to the following question: “In the past 30 days, have you consumed 5 (for men) or 4 (for women) or more doses of an alcoholic drink on one single occasion?” Those not indicating a minimum of 150 minutes of physical activity of moderate-intensity (or an equivalent of 75 minutes of vigorous activity) per week based on the following questions were classified as people with insufficient leisure-time physical activity (<150 minutes/week): “In the last three months, have you practiced any type of physical exercise or sport?”, “How many days a week do you practice physical exercise or sport?”, “On the day that you practice physical exercise or sport, how long does this activity last?”, and “What type of physical exercise or sport did you practice?”

The following sociodemographic characteristics of respondents were also used in analyses: sex (male and female), age group (18–34, 35–59, and ≥60 y), and years of schooling (0–8, 9–11, and ≥12 years).

From 2009 through 2019, 567,336 adults were interviewed by Vigitel. We first described the study population for each year according to sociodemographic characteristics. We used Prais–Winsten linear regression models to identify temporal variations among the indicators. We used item response theory (IRT) to analyze the coexistence of NCD behavioral risk factors. We chose to use the 2-parameter IRT model based on the Akaike information criterion statistical test. The length of the IRT premises (unidimensionality and local independence of item) was verified through the exploratory factorial analysis (a single factor explained 26.2% of the total variation of the answers) (14). We used all the behavioral risk behaviors to build the coexistence indicator. The assessment of a person’s capacity to have the behavioral risk factors simultaneously was based on the parameters of item discrimination (α) and on the item difficulty (β) indicated in the function applied to the IRT (12), allowing one to infer which indicators have a greater influence on the analyzed outcome (θ) (12). The evaluation of the coexistence of risk behavior indicators was complemented by the analysis of the test information curve (TIC) (12).

The latent variable values obtained in the IRT were multiplied by the standard deviation of the original score (0 to 5 behaviors) and were then added to the average of the original score (15). The latent variable values represent the count of behavioral risk factors accumulated by the person. We applied Poisson regression models to calculate crude and adjusted prevalence ratios (aPRs), adopting the score as a dependent variable, with the sociodemographic variables (sex, age, education) and the years of the study (time points) as explicative variables.

We used Stata software version 16.1 (StataCorp LLC) to organize and analyze the data. A significance level of 5% was adopted for all analyses. Vigitel’s databases are available for public use on the official website of the Brazil Ministry of Health (http://svs.aids.gov.br/download/Vigitel). Data collection was authorized by the National Commission of Ethics in Research for Human Beings of the Ministry of Health (no. 65610017.1.0000.0008).

## Results

During the study period, the age and education level of the adult population in Brazil increased. Thus, participation of adults aged 18 to 34 years decreased (42.9% to 38.8%), while the prevalence of other age groups increased, especially adults aged 60 years or older (14.7% to 18.3%). The percentage of adults with 0 to 8 years of schooling decreased (42.0% to 28.8%), while the percentage of adults with 12 years and more increased (22.2% to 32.8%) (Table 1).

**Table 1:** Sociodemographic Characteristics of the Adult Population in Brazil, Vigitel, 2009–2019^a^

Variable	Distribution of the adult population, %											Annual variation 2009–2019, %^b^ (95%CI) [*P* value]
	2009	2010	2011	2012	2013	2014	2015	2016	2017	2018	2019	
**Sex**												
Male	46.1	46.1	46.1	46.1	46.1	46.1	46.0	46.0	46.0	46.0	46.0	−0.03 (−0.03 to −0.02) [<.001]
Female	53.9	53.9	53.9	53.9	53.9	53.9	54.0	54.0	54.0	54.0	54.0	0.02 (0.02 to 0.03) [<.001]
**Age group, y**												
18–34	42.9	42.5	42.1	41.6	41.3	40.8	40.4	40.0	39.6	39.2	38.8	−1.00 (−1.01 to −0.99) [<.001]
35–59	42.4	42.3	42.5	42.8	42.6	42.9	42.7	42.9	42.8	42.7	42.9	0.11 (0.04 to 0.18) [.007]
≥60	14.7	15.2	15.4	15.6	16.1	16.3	16.9	17.1	17.6	18.1	18.3	2.19 (2.03 to 2.36) [<.001]
**Years of schooling**												
0–8	42.0	40.6	38.8	36.8	36.6	35.9	34.6	32.5	30.8	30.2	28.8	−3.68 (−4.07 to −3.28) [<.001]
9–11	35.8	35.8	36.7	38.5	37.5	38.1	38.1	35.9	37.3	38.0	38.4	0.48 (−0.16 to 1.12) [.01]
≥12	22.2	23.5	24.5	24.7	25.9	25.9	27.3	31.6	31.9	31.8	32.8	4.05 (3.07 to 5.04) [<.001]
**Total**	54,367	54,339	54,144	45,448	52,929	40,853	54,174	53,21	53,034	52,395	52,443	—

Abbreviation: —, does not apply.

a Data source: Surveillance of Risk and Protective Factors for Chronic Diseases Telephone Survey (Vigitel) (13).

b Corresponding to the Prais-Winsten regression coefficient of the variable over the survey year (expressed in percentage per year).

The most prevalent behavioral risk factors were infrequent consumption of fruits and vegetables (65.4%) and insufficient leisure-time physical activity (65.0%), while the least prevalent was smoking (11.4%). Behavioral risk factors that most contributed to the occurrence of coexistence of risk factors were smoking (α = 1.211), regular consumption of sugar-sweetened beverages (α = 0.879), and infrequent consumption of fruits and vegetables (α = 0.847). The behavioral risk factor that least contributed to coexistence was insufficient leisure-time physical activity (α = 0.344) (Table 2). The behavioral risk factors that indicated a greater capacity of people to have multiple risk behaviors were abusive alcohol consumption (β = 2.321), smoking (β = 2.106), and the regular consumption of sugar-sweetened beverages (β = 1.754). The behavioral risk factors that indicated a decrease in the capacity of people to have multiple risk behaviors were insufficient leisure-time physical activity (β = −1.850) and infrequent consumption of fruits and vegetables (β = −0.868) (Table 2). The TIC confirmed the test’s capacity to demonstrate the presence of the coexistence of risk behaviors for those with a greater accumulation of risk behaviors (Figure).

**Table 2:** Estimates and Classification of Parameters for Measuring the Coexistence of Risk Behaviors for Noncommunicable Diseases Among Adults in Brazil, 2009–2019^a^

Variable	Estimate, % (95% CI)	Item discrimination,^b^ α (95% CI)^c^	Classification^b^	Item difficulty, β (95% CI)^c^
Infrequent consumption of fruits and vegetables	65.4 (65.2 to 65.7)	0.847 (0.765 to 0.928)	Moderate discrimination	−0.868 (−0.933 to −0.803)
Regular consumption of sugar-sweetened beverages	20.8 (20.5 to 21.0)	0.879 (0.816 to 0.942)	Moderate discrimination	1.754 (1.655 to 1.853)
Smoking	11.4 (11.2 to 11.6)	1.211 (1.076 to 1.345)	Moderate discrimination	2.106 (1.948 to 2.263)
Abusive alcohol consumption	17.9 (17.6 to 18.1)	0.725 (0.642 to 0.808)	Moderate discrimination	2.321 (2.098 to 2.545)
Insufficient leisure-time physical activity	65.0 (64.7 to 65.3)	0.344 (0.301 to 0.386)	Low discrimination	−1.850 (−2.065 to −1.635)

a Data source: Surveillance of Risk and Protective Factors for Chronic Diseases Telephone Survey (Vigitel) (13).

b Item discrimination classification according to Baker (16).

c All values are significant at *P* < .001.

**Figure:**
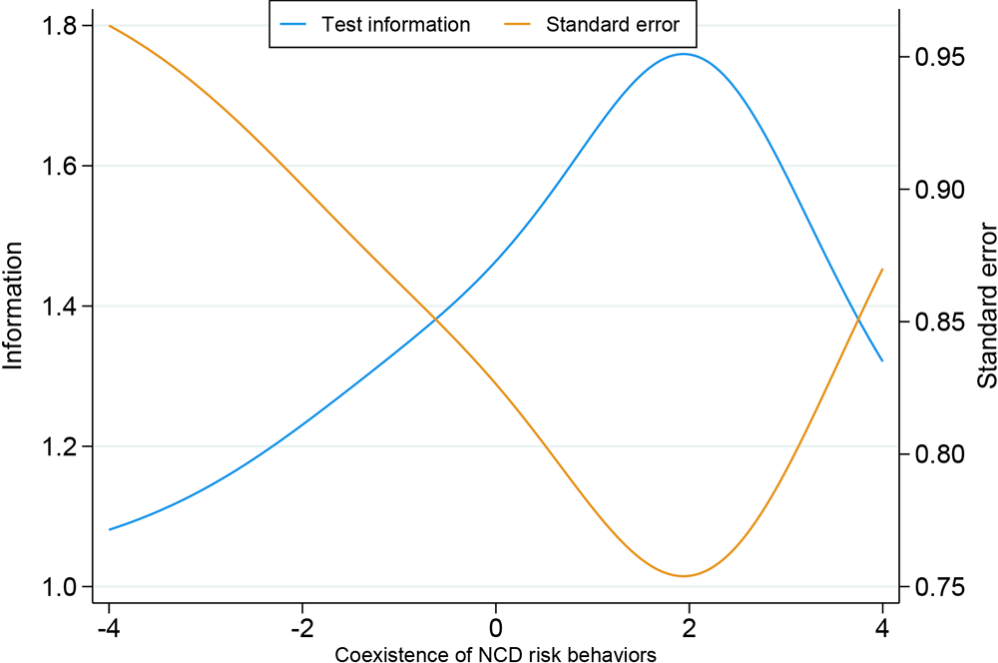
Test information curve of the measure of coexistence of noncommunicable disease–related risk behaviors among Brazilian adults. Data source: Surveillance System for Risk Factors and Protection for Chronic Diseases by Telephone Survey (Vigitel), 2009–2019. Abbreviation: NCD, noncommunicable disease.

The coexistence of risk behaviors was directly associated with male sex (11% higher for men than for women [aPR = 1.11; *P* < .001]), with younger age groups (14% higher among adults aged 35–59 y [aPR = 1.14; *P* < .001] and 21% higher among adults aged 18–34 y [aPR = 1.21; *P* < .001] than among adults age ≥60 y) and with lower schooling levels (8% higher among adults with 9–11 years of schooling [aPR = 1.08; *P* < .001] and 15% higher in those with 0–8 years of education [aPR = 1.15; *P* < .001] than among adults with 12 or more years of schooling). The prevalence of coexistence during the study period declined, with values 1% lower in 2012 (aPR = 0.99; *P* = .002) and 6% lower in 2015 (aPR = 0.94; *P* < .001). However, we also observed stagnation from 2016 to 2019 (Table 3). Among men, the coexistence of risk behaviors was 15% higher among men aged 35 to 59 years (aPR = 1.15; *P* < .001) and 19% higher among men aged 18 to 34 years (aPR = 1.19; *P* < .001) than among men aged 60 years or older. Similarly, among women, the coexistence of risk behaviors was higher among younger women: it was13% higher (aPR = 1.13; *P* < .001) among women aged 35 to 59 years and 23% higher among women aged 18 to 34 years than among women aged 60 years or older. We found a higher prevalence of coexistence among both men and women with lower levels of education. Among men, the aPR was 1.07 (*P* < .001) among those with 9 to 11 years of education and 1.16 (*P* < .001) among those with 0 to 8 years of education. Among women, the aPR was 1.09 (*P* < .001) among those with 9 to 11 years of education and 1.15 (*P* < .001) among those with 0 to 8 years of education. During the study period, we observed a decrease in the coexistence of risk behaviors among men and women similar to that observed among the total population (Table 3).

**Table 3:** Crude and Adjusted Prevalence Ratios (95% CI) of Coexistence of Risk Behaviors for Noncommunicable Diseases Among Adults in Brazil, by Sex, 2009–2019^a^

Variable	Total		Male		Female	
	Crude	Adjusted^b^	Crude	Adjusted^b^	Crude	Adjusted^b^
**Sex**						
Female	Reference	Reference	—	—	—	—
Male	1.13^c^ (1.12–1.13)	1.11^c^ (1.11–1.12)	—	—	—	—
**Age group, y**						
35–59	1.08^c^ (1.08–1.09)	1.14^c^ (1.13–1.14)	1.13 (1.13–1.14)	1.15^c^ (1.14–1.16)	1.10^c^ (1.09–1.10)	1.13^c^ (1.12–1.13)
18–34	1.11^c^ (1.10–1.11)	1.21^c^ (1.20–1.22)	1.14 (1.13–1.15)	1.19^c^ (1.18–1.20)	1.17^c^ (1.16–1.17)	1.23^c^ (1.22–1.24)
≥60	Reference	Reference	Reference	Reference	Reference	Reference
**Years of schooling**						
9–11	1.12^c^ (1.11–1.12)	1.08^c^ (1.07–1.08)	1.08 (1.07–1.09)	1.07^c^ (1.06–1.16)	1.08^c^ (1.08–1.09)	1.09^c^ (1.08–1.09)
0–8	1.16^c^ (1.16–1.17)	1.15^c^ (1.15–1.16)	1.12 (1.11–1.13)	1.16^c^ (1.15–1.17)	1.08^c^ (1.08–1.09)	1.15^c^ (1.14–1.16)
≥12	Reference	Reference	Reference	Reference	Reference	Reference
**Year**						
2009	Reference	Reference	Reference	Reference	Reference	Reference
2010	1.00 (0.99–1.01)	1.00 (1.00–1.01)	0.99 (0.98–1.01)	1.00 (0.98–1.01)	1.01 (1.00–1.01)	1.01 (1.00–1.02)
2011	0.99^d^ (0.98–0.99)	0.99 (0.98–1.00)	0.99 (0.98–1.00)	0.99 (0.98–1.01)	0.99^d^ (0.98–1.00)	0.99 (0.98–1.00)
2012	0.98^c^ (0.97–0.99)	0.99^d^ (0.98–0.99)	0.99^d^ (0.97–1.00)	0.99 (0.98–1.01)	0.97^c^ (0.96–0.98)	0.98^d^ (0.97–0.99)
2013	0.95^c^ (0.94–0.96)	0.96^c^ (0.95–0.97)	0.95^c^ (0.94–0.97)	0.96^c^ (0.95–0.97)	0.95^c^ (0.94–0.96)	0.96^c^ (0.95–0.97)
2014	0.94^c^ (0.93–0.95)	0.95^c^ (0.94–0.96)	0.94^c^ (0.94–0.97)	0.95^c^ (0.93–0.96)	0.94^c^ (0.93–0.95)	0.95^c^ (0.94–0.96)
2015	0.92^c^ (0.92–0.93)	0.94^c^ (0.93–0.94)	0.92^c^ (0.91–0.94)	0.93^c^ (0.92–0.95)	0.92^c^ (0.91–0.94)	0.94^c^ (0.93–0.95)
2016	0.93^c^ (0.92–0.94)	0.95^c^ (0.94–0.95)	0.93^c^ (0.91–0.94)	0.94^c^ (0.93–0.95)	0.93^c^ (0.92–0.94)	0.95^c^ (0.94–0.96)
2017	0.92^c^ (0.92–0.93)	0.94^c^ (0.93–0.95)	0.93^c^ (0.91–0.94)	0.95^c^ (0.93–0.96)	0.92^c^ (0.91–0.93)	0.94^c^ (0.93–0.95)
2018	0.92^c^ (0.91–0.93)	0.94^c^ (0.93–0.95)	0.92^c^ (0.91–0.93)	0.94^c^ (0.92–0.95)	0.92^c^ (0.91–0.93)	0.94^c^ (0.93–0.95)
2019	0.92^c^ (0.91–0.93)	0.94^c^ (0.94–0.95)	0.92^c^ (0.91–0.93)	0.94^c^ (0.92–0.95)	0.93^c^ (0.92–0.94)	0.95^c^ (0.94–0.96)

Abbreviation: —, does not apply.

a Data source: Surveillance of Risk and Protective Factors for Chronic Diseases Telephone Survey (Vigitel) (13).

b Adjusted for sex, age group, years of schooling, and year. *P* value estimated by Poisson regression analysis.

c *P* < .001.

d *P* < .05.

## Discussion

Data collected from more than 560,000 adults in Brazil during more than a decade (2009–2019) enabled a comprehensive study of the coexistence of NCD risk behaviors and allowed the analysis of their temporal evolution. The risk factors that most consistently contributed to the coexistence of NCD risk behaviors were smoking, regular consumption of sugar-sweetened beverages, and abusive alcohol consumption (with moderate discrimination between the item and the high capacity to have the behavior among adults with multiple risk behaviors). The coexistence of these behaviors was greater among men than women and was inversely associated with age group and years of schooling. During the study period, we observed a decrease in the coexistence of NCD risk behaviors, with a stabilization from 2015 onward (4%–5% per year). This decrease remained significant after adjustment for sex, age, and years of schooling.

In general, the results of our study expand and update the findings from other investigations conducted among the adult population in Brazil. The first study, which used multivariate techniques, was conducted with data collected during 2009 and 2010 among adults residing in Brazilian capitals (N = 108,706); it aimed to identify patterns of NCD protective and risk factors through principal component analysis. Two behavior patterns were identified: a “prudent pattern” and a “risky pattern.” The least healthy patterns were concentrated among younger men with lower levels of education (17). Another study with data from a household study representative of the Brazilian population in 2013 (N = 46,785 adults aged ≥20 y) used a cluster analysis and observed that the grouping of risk behaviors was more common among men, those with a lower education level, and among Black and Brown individuals (18).

Until our study, the only other investigation aiming to analyze the temporal trend in the coexistence of NCD-related behaviors focused on protective behaviors and used data from 2008 through 2013 (11). The study estimated the prevalence of the coexistence of healthy behaviors and its association with education level (N = 200,000 adults) and found that the prevalence of the coexistence of healthy behaviors increased and was directly associated with education level (11). However, the comparison with our findings requires caution because of differences in the databases (19) and analytic approaches used. Nevertheless, both analyses found similar effects in the coexistence of NCD-related behaviors on their temporal trend, observing a favorable evolution (11).

The greater frequency of risk behaviors among men is recurrent in national surveys (13,20). Many of these behaviors may be related to concepts culturally constructed in society, such as exposing oneself to risks that reinforce masculinity or to lower levels of access to health information caused by lower self-demand for medical care (21). Exposure to risky behaviors might be more common among younger populations because older populations are more concerned about health and the emergence of diseases or injuries leads to the adoption of healthier habits (4). In addition, groups with higher education levels may have the lowest risk of coexisting risk behaviors, probably because of access to a better quality of life and better access to information and health services, factors positively associated with the adoption of healthy habits (11). In this context, it becomes essential to strengthen health education actions for groups with a lower level of education to prevent adoption of NCD risk behaviors.

Even though the prevalence of the coexistence of NCD risk factors decreased overall during the study period, we found stagnation in the more recent segment (especially after 2015). This scenario possibly reflects the weakening in health promotion policies that directly affect risk behaviors (such as stabilization in the prevalence of smoking) (8). In parallel, Brazil experienced the degradation of many quality-of-life–related economic indicators, such as the Gini and Palma indexes, from 2012 through 2019 (20), and the health indicators established in the *Strategic Action Plan to Tackle Noncommunicable Diseases (NCD) in Brazil, 2011–2022* (21,22). Questions concerning the funding of the Brazilian Unified Health System were raised by the government’s freezing of health expenses in 2010 and the imposition of austerity measures in 2016 (23,24).

Although this scenario — the coexistence of risk behaviors — has worsened, the prevalence of risk behaviors has decreased (5–7), driven by an increase in access to health services and a structural improvement in living conditions (25). These factors may be reflected in significant increases in life expectancy from 2010 to 2019 (among men, from 70.2 to 73.1 y, and among women, from 77.6 to 80.1 y) (25). Consequently, the population in Brazil is aging, and the rate of premature death has been substantially reduced (25), resulting in a constant increase in the absolute burden of diseases (mainly NCDs) and its effect on health systems (26). Such a scenario reinforces the need for a strict surveillance of the trends in health indicators, as well as the actions to stop or revert those that are evolving negatively (26).

The implementation of effective public policies targeting NCDs and their risk behaviors is essential. In Brazil, the National Health Promotion Policy, implemented in 2006, outlines the guidelines aimed at equitably improving the health conditions of the population, and risk factors are priority topics (27). The update of the strategic action plan to tackle chronic diseases defines the necessary actions to control NCDs until 2030 (28). Most of the actions proposed in the updated strategic plan are similar to those in the original plan (22). However, several proposals in the original version were not adequately implemented or not implemented at all during the term of the original plan (2011–2022) and as a result, proposed goals were not achieved (22,28). Although the overall coexistence of NCD behavioral risk factors decreased during our study period, previous research on isolated NCD risk and protective factors (28) indicates fragility in implemented policies. For example, several actions related to alcohol consumption (such as zero tolerance to drinking and driving or inspection of sales to people younger than 18 years) were informally relaxed after implementation, which decreased the effect on alcohol consumption (28). In addition to the actions outlined by the strategic plan, other publications, programs, and policies, such as the Dietary Guidelines for the Brazilian Population (29), Programa Academia da Saúde (Health Academy Program) (30), a framework for tobacco control (31), and a national alcohol policy (32) aim to curb the prevalence of NCD behavioral risk factors.

Effective public policies for reducing NCD risk behaviors that account for the coexistence of these behaviors should be prioritized in Brazil. The following factors should be considered in selecting interventions for preventing and controlling NCDs: efficacy, cost-effectiveness, accessibility, capacity for implementation, feasibility, impact on health equity, and a combination of policies at the collective and individual levels (33). Interventions based on the theory of social learning or focusing on multiple risk behaviors can also help to improve health outcomes. This type of intervention aims to work with multiple interactions among health behaviors, aiming at healthier lifestyles and reducing risk behaviors, and can maximize the effect on populations at high risk of NCDs and in need of lifestyle changes. However, it still needs to better clarify the effect of joint action in relation to NCDs (34,35).

Although the literature identifies the coexistence of NCD risk behaviors and its association with sociodemographic characteristics, little progress has been made concerning the factors that most commonly influence this coexistence. In this context, we note the analytic approach used in this study. The IRT, still rarely used in epidemiology, differs from other traditional methods (such as cluster analysis and principal component analysis), enabling improvement in the quality of the estimates based on epidemiologic questionnaires (in which variables are rarely distributed normally) (36). Moreover, the method makes it possible to view which behaviors have a greater influence on the model (14).

### Limitations

Some limitations should be considered. First is the use of self-reported data by Vigitel; self-reported data can be less accurate than directly observed data. However, self-reported information is frequently used in large health surveys performed by telephone interviews (37,38), and the reproducibility and validity of the health indicators available in Vigitel have been reported in recent studies (39,40). Second, the sample was restricted to adults with a landline residing in a Brazilian capital; this limitation is minimized by weighting factors that allow extrapolation of results to the total population (13). Third, although we used relevant indicators for the main NCD risk factors, we did not use all indicators for these behaviors or even other relevant behaviors (such as screen time, smoking, frequency of consumption of alcoholic beverages). Such additional indicators could be used in future studies, expanding knowledge about the coexistence of NCD risk factors.

Despite these limitations, our study has strengths. In addition to being the first nationwide study to investigate the temporal evolution of the coexistence of NCD risk behaviors in a population of more than 500,000 adults in Brazil, our study stands out by presenting which risk behaviors, among those analyzed, have the highest possibility of occurring when the coexistence of risk behaviors in the population is observed.

### Conclusion

Our study identified a reduction in the frequency of the coexistence of NCD risk behaviors in Brazil from 2009 through 2019, especially in the first half of the study period. The association of sociodemographic characteristics with the coexistence of these risk behaviors was observed, which reinforces the need to advance public policies for the population groups most vulnerable to NCDs and to implement strategies to tackle the coexistence of NCD risk behaviors.

## References

[1] World Health Organization. Noncommunicable Diseases (NCD) country profiles. 2018. Geneva (CH): World Health Organization. Accessed May 20, 2021. https://apps.who.int/iris/handle/10665/274512

[2] Stanaway JD , Afshin A , Gakidou E , Lim SS , Abate D , Abate KH , ; GBD 2017 Risk Factor Collaborators. Global, regional, and national comparative risk assessment of 84 behavioural, environmental and occupational, and metabolic risks or clusters of risks for 195 countries and territories, 1990–2017: a systematic analysis for the Global Burden of Disease Study 2017. Lancet 2018;392(10159):1923–94. 10.1016/S0140-6736(18)32225-6 30496105 PMC6227755

[3] Cortina-Borja M , Smith AD , Combarros O , Lehmann DJ . The synergy factor: a statistic to measure interactions in complex diseases. BMC Res Notes 2009;2(1):105. 10.1186/1756-0500-2-105 19527493 PMC2706251

[4] Nyberg ST , Singh-Manoux A , Pentti J , Madsen IEH , Sabia S , Alfredsson L , Association of healthy lifestyle with years lived without major chronic diseases. JAMA Intern Med 2020;180(5):760–8. 10.1001/jamainternmed.2020.0618 32250383 PMC7136858

[5] Figueiredo N , Maia EG , Silva LESD , Granado FS , Claro RM . Trends in sweetened beverages consumption among adults in the Brazilian capitals, 2007-2016. Public Health Nutr 2018;21(18):3307–17. 10.1017/S1368980018002161 30207262 PMC10261081

[6] Cruz MSD , Bernal RTI , Claro RM . Tendência da prática de atividade física no lazer entre adultos no Brasil (2006–2016) [Trends in leisure-time physical activity in Brazilian adults (2006–2016)]. Cad Saude Publica 2018;34(10):e00114817. 10.1590/0102-311x00114817 30365744

[7] Silva LESD , Claro RM . Tendências temporais do consumo de frutas e hortaliças entre adultos nas capitais brasileiras e Distrito Federal, 2008-2016 [Time trends in the consumption of fruits and vegetables among adults in Brazilian state capitals and the Federal District, 2008–2016]. Cad Saude Publica 2019;35(5):e00023618. 10.1590/0102-311x00023618 31116246

[8] Maia EG , Stopa SR , de Oliveira Santos R , Claro RM . Trends in prevalence of cigarette smoking in Brazil (2006–2019). Am J Public Health 2021;111(4):730–8. 10.2105/AJPH.2020.306102 33600255 PMC7958007

[9] Granado FS , Maia EG , Mendes LL , Claro RM . Reduction of traditional food consumption in Brazilian diet: trends and forecasting of bean consumption (2007–2030). Public Health Nutr 2021;24(6):1185–92. 10.1017/S1368980020005066 33314999 PMC10195620

[10] Flores-Ortiz R , Malta DC , Velasquez-Melendez G . Adult body weight trends in 27 urban populations of Brazil from 2006 to 2016: a population-based study. PLoS One 2019;14(3):e0213254. 10.1371/journal.pone.0213254 30840675 PMC6402686

[11] Camelo LV , de Figueiredo RC , Oliveira-Campos M , Giatti L , Barreto SM . Comportamentos saudáveis e escolaridade no Brasil: tendência temporal de 2008 a 2013 [Healthy behavior patterns and levels of schooling in Brazil: time trend from 2008 to 2013]. Cien Saude Colet 2016;21(4):1011–21. 10.1590/1413-81232015214.09742015 27076000

[12] Pasquali L . TRI – Teoria de resposta ao item: teoria, procedimentos e aplicações. Curitiba (PR): APPRIS; 2018.

[13] Ministério da Saúde. Vigitel Brasil 2019: vigilância de fatores de risco e proteção para doenças crônicas por inquérito telefônico [Vigitel Brazil 2019: surveillance of risk and protective factors for chronic diseases by telephone survey]. Brasília (DF): Ministério da Saúde; 2020.

[14] Couto G , Primi R . Teoria de resposta ao item (TRI): conceitos elementares dos modelos para itens dicotômicos [Item response theory (IRT): elementary concepts of models for dichotomous items]. Bol Psicol 2011;61(134):1–15.

[15] Castro SMJ , Trentini C , Riboldi J . Teoria da resposta ao item aplicada ao Inventário de Depressão Beck [Item response theory applied to the Beck Depression Inventory]. Rev Bras Epidemiol 2010;13(3):487–501. 10.1590/S1415-790X2010000300012 20857035

[16] Baker FB . The basics of item response theory. 2nd ed. Washington (DC): Eric Clearinghouse on Assessment and Evaluation; 2001.

[17] Steele EM , Claro RM , Monteiro CA . Behavioural patterns of protective and risk factors for non-communicable diseases in Brazil. Public Health Nutr 2014;17(2):369–75. 10.1017/S1368980012005472 23308392 PMC10282382

[18] Silva DA , Rinaldi AEM , Azeredo CM . Clusters of risk behaviors for noncommunicable diseases in the Brazilian adult population. Int J Public Health 2019;64(6):821–30. 10.1007/s00038-019-01242-z 31062035

[19] Ministério da Saúde. Vigitel Brasil 2012: vigilância de fatores de risco e proteção para doenças crônicas por inquérito telefônico [Vigitel Brazil 2012: protective and risk factors for chronic diseases by telephone survey]. Brasília (DF): Ministério da Saúde; 2013. Accessed January 4, 2022. https://bvsms.saude.gov.br/bvs/publicacoes/vigitel_brasil_2012_vigilancia_risco.pdf

[20] Instituto Brasileiro de Geografia e Estatística. Síntese de indicadores sociais: uma análise das condições de vida da população brasileira: 2020 [Synthesis of social indicators: an analysis of the living conditions of the Brazilian population]. IBGE; 2020.

[21] Malta DC , Silva AG , Teixeira RA , Machado IE , Coelho MRS , Hartz AM . Avaliação do alcance das metas do plano de enfrentamento das doenças crónicas não transmissíveis no Brasil, 2011-2022 [Evaluation of the achievement of the goals of the Strategic Action Plan for Coping with Chronic Diseases in Brazil, 2011–2022]. An Inst Hig Med Trop (Lisb) 2019;1:9–16. 10.25761/anaisihmt.316

[22] Ministério da Saúde. Plano de ações estratégicas para o enfrentamento das doenças crônicas não transmissíveis (DCNT) no Brasil, 2011–2022 [Strategic Action Plan to Tackle Noncommunicable Diseases (NCD) in Brazil, 2011–2022]. Brasília, DF: Ministério da Saúde; 2011.

[23] Emenda Constitucional n. 95. Altera o Ato das Disposições Constitucionais Transitórias, para instituir o Novo Regime Fiscal [Constitutional Amendment no. 95. Amends the Temporary Constitutional Provisions Act, to institute the New Fiscal Regime.]. Brasília; 2016.

[24] Rossi P , Dweck E . Impactos do novo regime fiscal na saúde e educação [Impacts of the new tax regime on health and education]. Cad Saude Publica 2016;32(12):e00194316. 10.1590/0102-311x00194316 27992044

[25] Instituto Brasileiro de Geografia e Estatística. Tábua completa de mortalidade para o Brasil – 2019. Breve análise da evolução da mortalidade no Brasil [Complete life tables for Brazil — 2019. Brief analysis of the evolution of mortality in Brazil]. IBGE; 2020.

[26] Vos T , Lim SS , Abbafati C , Abbas KM , Abbasi M , Abbasifard M , ; GBD 2019 Diseases and Injuries Collaborators. Global burden of 369 diseases and injuries in 204 countries and territories, 1990-2019: a systematic analysis for the Global Burden of Disease Study 2019. Lancet 2020;396(10258):1204–22. 10.1016/S0140-6736(20)30925-9 33069326 PMC7567026

[27] Ministério da Saúde. Portaria no. 2.446, de 11 de novembro de 2014. Redefine a Política Nacional de Promoção da Saúde (PNPS) [Ordinance no. 2446, of November 11, 2014. Redefines the National Health Promotion Policy]. Brasília, DF: Ministério da Saúde; 2014.

[28] Ministério da Saúde. Plano de Ações Estratégicas para o enfrentamento das doenças crônicas e agravos não transmissíveis no Brasil 2021–2030 [Strategic actions plan for tackling chronic diseases and noncommunicable diseases in Brazil 2021–2030]. Brasília, DF: Ministério da Saúde; 2021.

[29] Ministério da Saúde. Guia alimentar para a população brasileira [Dietary guidelines for the Brazilian population]. Brasília, DF: Ministério da Saúde, 2014.

[30] Ministério da Saúde. Portaria no. 2.681, de 7 de novembro de 2013. Redefine o Programa Academia da Saúde no âmbito do Sistema Único de Saúde (SUS) [Ordinance no. 2681, of November 7, 2013. Redefines the Health Gym Program within the Unified Health System (SUS)]. Brasília, DF: Ministério da Saúde, 2013.

[31] Instituto Nacional de Câncer José Alencar Gomes da Silva. Convenção-quadro para controle do tabaco: texto oficial [Framework convention on tobacco control]. Rio de Janeiro, RJ: INCA, 2015.

[32] Presidência da República. Decreto no. 6.117, de 22 de maio de 2007. Aprova a Política Nacional sobre o Álcool, dispõe sobre as medidas para redução do uso indevido de álcool e sua associação com a violência e criminalidade, e dá outras providências [Decree no. 6,117, of May 22, 2007. Approves the national policy on alcohol, provides for measures to reduce the misuse of alcohol and its association with violence and crime, and other measures]. Publicada em: 22 de maio de 2007. DOU de 23.5.2007. Brasília, DF; 2007.

[33] World Health Organization. Tackling NCDs — “best buys” and other recommended interventions for the prevention and control of noncommunicable diseases. 2017. Accessed May 20, 2021. https://apps.who.int/iris/handle/10665/259232

[34] Toobert DJ , Glasgow RE , Strycker LA , Barrera M Jr , Ritzwoller DP , Weidner G . Long-term effects of the Mediterranean lifestyle program: a randomized clinical trial for postmenopausal women with type 2 diabetes. Int J Behav Nutr Phys Act 2007;4(1):1–12. 10.1186/1479-5868-4-1 17229325 PMC1783667

[35] Prochaska JJ , Prochaska JO . A review of multiple health behavior change interventions for primary prevention. Am J Lifestyle Med 2011;5(3):208–21. 10.1177/1559827610391883 24358034 PMC3865280

[36] Gorter R , Fox JP , Twisk JWR . Why item response theory should be used for longitudinal questionnaire data analysis in medical research. BMC Med Res Methodol 2015;15:55. 10.1186/s12874-015-0050-x 26224012 PMC4520067

[37] Riley L , Guthold R , Cowan M , Savin S , Bhatti L , Armstrong T , The World Health Organization STEPwise approach to noncommunicable disease risk-factor surveillance: methods, challenges, and opportunities. Am J Public Health 2016;106(1):74–8. 10.2105/AJPH.2015.302962 26696288 PMC4695948

[38] Pickens CM , Pierannunzi C , Garvin W , Town M . Surveillance for certain health behaviors and conditions among states and selected local areas — Behavioral Risk Factor Surveillance System, United States, 2015. MMWR Surveill Summ 2018;67(9):1–90. 10.15585/mmwr.ss6709a1 29953431 PMC6023179

[39] Ferreira AD , César CC , Malta DC , Andrade AC , Ramos CG , Proietti FA , Validade de estimativas obtidas por inquérito telefônico: comparação entre VIGITEL 2008 e inquérito Saúde em Beagá [Validity of data collected by telephone survey: a comparison of VIGITEL 2008 and ‘Saúde em Beagá’ survey]. Rev Bras Epidemiol 2011;14(Suppl 1):16–30. 10.1590/S1415-790X2011000500003 22002139

[40] Caldeira TCM , Soares MM , Silva LESD , Veiga IPA , Claro RM . Comportamentos de risco e proteção para doenças crônicas nas capitais brasileiras e no Distrito Federal, segundo a Pesquisa Nacional de Saúde e o Sistema de Vigilância de Fatores de Risco e Proteção para Doenças Crônicas por Inquérito Telefônico, 2019 [Chronic disease risk and protective behaviors in Brazilian state capitals and the Federal District, according to the National Health Survey and the Chronic Disease Risk and Protective Factors Telephone Survey Surveillance System, 2019]. Epidemiol Serv Saude 2022;31(Suppl 1):e2021367. 10.1590/ss2237-9622202200009.especial 35946669 PMC9897819

